# See (n)One, Do (n)One, Teach (n)One: Reality of Surgical Resident Training in Germany

**DOI:** 10.1007/s00268-020-05539-6

**Published:** 2020-04-30

**Authors:** T. Huber, I. Richardsen, C. Klinger, M. Mille, A. A. Roeth, the AsTerOiD-Study Group, Johannes Dörner, Sebastian Drewes, Stefan Fabig, Jochen Gaedcke, Patricia J. Günter, Dominik Jauch, Franziska Koch, Jana Lenzen, Marianne Obst, Ulrike Orthaus, Vivien Schacke, Christian von Schassen, Laura Scheurer, Sophia Schmitz, Ben Scholtes, Johanna Schreier, Nils P. Sommer, Dirk Uhlmann, Maria Wobith, Michael Zaczek

**Affiliations:** 1 Young Surgeons Working Group (CAJC) of the German Society for General and Visceral Surgery (DGAV), Berlin, Germany; 2 Department of General, Visceral and Transplant Surgery, University Medical Center of the Johannes Gutenberg-University, Langenbeckstraße 1, 55131 Mainz, Germany; 3 Department of General, Visceral and Thoracic Surgery, German Armed Forces Central Hospital, Koblenz, Germany; 4 German Society for General and Visceral Surgery (DGAV), Berlin, Germany; 5 Department of General and Visceral Surgery, HELIOS Hospital Erfurt, Erfurt, Germany; 6 Department of General, Visceral and Transplant Surgery, RWTH Aachen University Hospital, Aachen, Germany

## Abstract

**Introduction:**

Due to technological changes, working time restrictions and the creation of specialized centers, surgical training has changed. A competence-based learning technique of surgical skills is the sub-step practice approach, which has been proven important in nationwide opinion surveys. The aim of this prospective multi-center trial was to determine the status quo of the sub-step concept in Germany.

**Methods:**

Over 6 months, the voluntarily participating centers evaluated the following index procedures: laparoscopic cholecystectomy (LCHE), laparoscopic and open sigmoid resection, minimally invasive inguinal hernia repair, thyroid resection and pylorus-preserving pancreaticoduodenectomy (PPPD). Patients with private insurance were excluded. The detailed sub-steps were documented as well as the reason why these were not performed. In addition, an online survey regarding the sub-step concept was performed before and after the study.

**Results:**

In total, 21 centers included 2969 surgical procedures in 2018 for final analyses. While 24.4% of the procedures were performed by residents, sub-steps were performed in 22.2%. LCHE was most often performed completely by residents (43.3%), and PPPD revealed the highest rate of performed sub-steps (43.3%). Reasons for not assisting sub-steps to residents were often organizational and other reasons. After an initial increase, the number of performed sub-steps decreased significantly during the second half of the survey. The opinion survey revealed a high importance of the sub-step concept. The number of resident procedures was overestimated, and the number of performed sub-steps was underestimated. After the study, these estimations were more realistic.

**Conclusion:**

Even though the sub-step practice concept is considered highly important for surgical education, it needs to be put into practice more consequently. The current data suggest a low participation of surgical residents in the operating room, although the participating hospitals are most likely highly interested in surgical education, hence their voluntary participation. Conceptual changes and a control of surgical education are needed.

**Electronic supplementary material:**

The online version of this article (10.1007/s00268-020-05539-6) contains supplementary material, which is available to authorized users.

## Introduction

The training of surgical skills has changed dramatically over the last decades. Technological advances with laparoscopic and robotic techniques enlarge the variety of skills a trainee needs to acquire [1]. The creation of specialized surgical centers limits the availability of basic general surgical procedures in specialized centers and of complex surgeries in primary care hospitals. Furthermore, the European working time directive reduces the time residents spend in the hospital and the operating room (OR) and Kaser et al. [2] further demand to better utilize surgical teaching opportunities and to re-structure surgical training. Surgical training needs to adapt to these changes, e.g., by using simulators to enable skill acquisition outside of the OR. Furthermore, board certification in Germany is still not competency based, but focusses only on a minimum number of performed procedures.

A competency-based approach has been proposed by the Young Surgeons Working Group (CAJC) of the German Society for General and Visceral Surgery (DGAV). On the one hand, surgeries have been categorized according to the postgraduate year (PGY), starting with appendectomy or hernia repair for PGY 1–2; cholecystectomy and hemithyroidectomy for PGY 3–4 and colon resections and fundoplication for PGY 5–6 [3]. On the other hand, a concept of surgical sub-steps was developed—based on the theoretical concept of DaRosa et al. [4]—describing a tool to acquire certain surgical skills as part of more complex operations that might be too difficult for the trainee at the time (e.g., laparotomy for a second year resident during a complex hepatic or pancreatic resection) [3, 5].

Nationwide questionnaire surveys among residents in Germany found that surgical sub-steps are performed in only 27% of departments. In contrast, surgical program directors estimate this percentage around 54%  [3, 6]. A first monocentric analysis revealed that this concept can be easily implemented in a surgical department and increases the number of performed sub-steps as well as resident satisfaction [7].

The aim of this prospective multi-center trial was to analyze the current status of the surgical sub-step concept in German general and visceral surgery departments.

## Methods

The study was conducted as an exploratory prospective survey in voluntarily participating centers for a total of 6 months (first patient–last patient). The starting point of the study differed in each center resulting in a time period from February to December 2018. The study was promoted beforehand for 9 months on local surgical conferences and courses in Germany as well as newsletters of the DGAV in 2017 and 2018. It was approved by the Ethics Committee of the State Chamber of Medicine in Rhineland-Palatinate. Since individual patient data were not recorded, informed patient consent was considered not necessary by the ethics committee.

Based on the sub-step practice approach of the CAJC [3], five index procedures for general and visceral surgery were defined and divided into sub-steps: laparoscopic cholecystectomy (LCHE), laparoscopic and open sigmoid resection (SIGR), minimally invasive inguinal hernia repair (MIHR), thyroid/hemithyroid resection (THYR) and pylorus-preserving pancreaticoduodenectomy (PPPD) (Table 1). These procedures were chosen since they reflect a selection of the board certification requirements (minimum number in parenthesis) in Germany: cholecystectomies (25), colon resections (10), hernia repair (25), surgeries of the neck (25), intraabdominal procedures in general (400), assistance during complex procedures (60).

**Table 1:** Index operations and detailed sub-steps

Procedure	Surgical sub-steps
Laparoscopic cholecystectomy (LCHE)	1. Access to the abdomen 2. Trocar placement and exploration 3. Preparation of Calot’s triangle 4. Clip application to cystic duct and artery 5. Gallbladder removal 6. Retrieval of gallbladder and closure
Sigmoid resection (open and laparoscopic) (SIGR)	1. Access to the abdomen 2. Mobilization of the sigmoid 3. Exposition of the ureter 4. Mobilization of the left flexure 5. Dissection of the mesentery/artery dissection 6. Preparation of the upper rectum 7. Stapling of the upper rectum 8. Mini laparotomy and evisceration 9. Resection of the sigmoid 10. Anastomosis 11. Abdominal closure
Hemithyroidectomy/thyroidectomy (THYR)	1. Access to the thyroid gland 2. Mobilization of the thyroid lobe 3. Preparation of vagal nerve for neuromonitoring 4. Preparation of recurrent nerve 5. Preparation of parathyroids 6. Resection of the thyroid lobe 7. Closure of access
Minimally invasive inguinal hernia repair (MIHR)	1. Access to the abdomen/preperitoneal area 2. Identification and reposition of hernia content 3. Preparation of the hernia sac 4. Application of hernia mesh 5. Peritoneal closure 6. Closure of access
Whipple’s operation/pylorus-preserving pancreaticoduodenectomy (PPPD)	1. Laparotomy 2. Opening of bursa omentalis 3. Preparation (Kocher’s maneuver) 4. Lymphadenectomy 5. Cholecystectomy 6. Resection of the pancreas 7. Pancreatico-jejunostomy/-gastrostomy 8. Bilio-digestive-anastomosis 9. Duodeno-/gastro-jejunostomy 10. Abdominal closure

For each participation center, one representative was selected (listed as collaborator). These have been instructed how to document the procedures anonymously in an online database using a personalized login. In a prospective manner, the collaborators interviewed the surgical teams on the intraoperative performance of sub-steps and also directly asked for reasons, why this was not done during a case. The type of procedure, the detailed sub-steps and the rank and extent of involvement of all team members were recorded. Furthermore, reasons why sub-steps or the whole procedure had not been performed by a resident were also registered using a free text field and the following categories:

- *patient risk factors* (comorbidities or anatomical and surgical risk factors such as extensive adhesions due to prior surgeries)
- *procedure too complex* (for the available resident, e.g., if the available resident is only in his first few years of training)
- *young surgical fellow performing the procedure (within 1 year after the German board examination for general or visceral surgery, which takes place after at least 6 years of residency)*
- *organizational reasons* (time pressure, logistics, no resident available who is suitable for the respected sub-step)
- *other reasons* (that do not match any of the reasons listed above)

Since patients with private insurance have the right to select their surgeon, these patients had to be excluded from the analysis. Centers that did not document the total of 6 months or included less than 20 surgical procedures in total were excluded from the final analysis to gain representative data.

A voluntary online survey regarding the knowledge about the sub-step practice approach, the general implementation in the department and the estimated percentage of performed sub-steps regarding the index operations was conducted in all participating centers. This was performed before and after the survey period.

For statistical analysis, IBM SPSS Statistics 23 (IBM. Armonk. NY. USA) was used. Data were exported from the online database tool as SPSS table sheet. Nonparametric Mann–Whitney-*U*-test and Fishers’ exact test were used for calculation. *p* values below 0.05 were considered statistically significant.

## Results

In total, 21 centers qualified for final analysis after application of the exclusion criteria. These included 7 university hospitals, 7 tertiary care centers, 5 secondary care centers and 2 primary care centers.

A total of 2969 surgical procedures were documented in the online database tool from February to December 2018. All centers reported a documentation of 100% regarding the performed index operations.

**Fig. 1:**
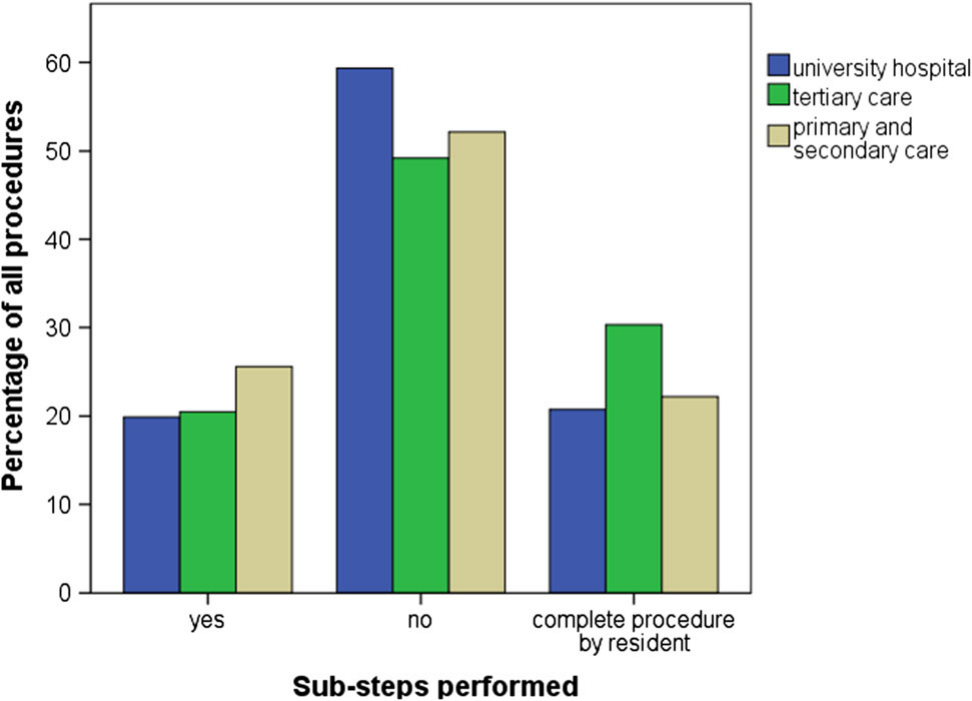
Overview of performed sub-steps and procedures performed by residents according to the care level of the participating center (university hospital: *n* = 7; tertiary care: *n* = 7; primary and secondary care: *n* = 7; *p* > 0.05)

Table 2 displays the surgical procedures primarily performed by residents (24.4% in total). Sub-steps were generally performed in 22.2% (29.4% of operations that were not performed by a resident completely). PPPD revealed the highest percentage of performed sub-steps. There was no correlation between the center’s care level of the participation center and number of assisted sub-steps or surgeries performed by residents (Fig. 1).

**Table 2:** Analysis of the documented surgical procedures

Procedure	*n*	Performed by resident *n* (%)	Sub-steps performed *n* (%)	No sub-steps performed *n* (%)
LCHE	1036	449 (43.3)	186 (18.0)	401 (38.7)
SIGR	217	17 (7.8)	55 (25.3)	145 (66.8)
THYR	788	85 (10.8)	171 (21.7)	532 (67.5)
MIHR	748	172 (23.0)	170 (22.7)	406 (54.3)
PPPD	180	0 (0)	78 (43.3)	102 (56.7)
Total	2969	723 (24.4)	660 (22.2)	1586 (53.4)

*LCHE* laparoscopic cholecystectomy, *SIGR* laparoscopic/open sigmoid resection, *THYR* thyroid resection, *MIHR* minimally invasive inguinal hernia repair, *PPPD* pylorus-preserving pancreaticoduodenectomy

**Fig. 2:**
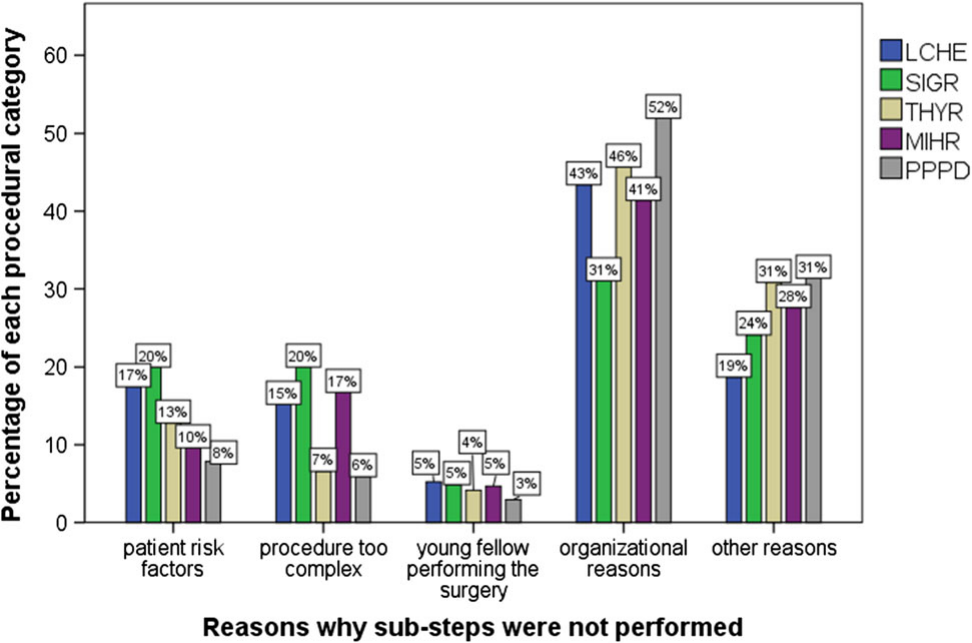
Reasons why sub-steps were not performed (young fellow: within 1 year of board examination)

Reasons why sub-steps were not performed are displayed in Fig. 2. Young fellows performed the surgery in about 5% of procedures regardless of the category. The analysis revealed a high percentage of *organizational reasons* and *other reasons*. A subgroup analysis of these *organizational reasons* revealed, that in 77.9% of these cases (*n* = 531/683) no resident was present during the operation.

LCHE was performed by residents in almost half of the cases (Table 3A). In 186 cases (18.0%), sub-steps were performed. Of these, access and closure of the abdomen were the most frequent sub-steps, while preparation and ligation of the cystic duct and the cystic artery were performed less frequently.

**Table 3:** A–E: Detailed analysis of procedures (A: LCHE, B: SIGR, C: THYR, D: MIHR, E: PPPD) in total and with respect to the involved residents’ postgraduate year (PGY)

	Postgraduate year							Total
	1	2	3	4	5	6	6+	
(A)								
Laparoscopic cholecystectomy (LCHE)	152	79	169	163	134	123	37	860
No resident present								176
Resident present but no involvement	83	33	42	29	20	14	4	225
Procedure performed by resident	24	24	82	97	93	98	31	449
Sub-steps performed	45	22	45	37	21	11	5	186
Access to the abdomen	21	19	42	31	18	9	4	144
Trocar placement and exploration	21	12	39	29	17	8	4	130
Preparation of Calot’s triangle	2	1	8	10	12	8	2	43
Clip application to cystic duct and artery	5	2	16	19	10	6	1	59
Gallbladder removal	16	4	28	19	11	6	1	85
Retrieval of gallbladder and closure	32	11	34	30	16	9	2	134

	Postgraduate year							Total
	1	2	3	4	5	6	6+	
(B)								
Sigmoid resection (SIGR)	29	12	21	29	39	28	9	167
No resident present								50
Resident present but no involvement	23	9	14	14	23	8	4	95
Procedure performed by resident	0	0	1	2	6	6	2	17
Sub-steps performed	6	3	6	13	10	14	3	55
Access to the abdomen	1	2	3	9	5	5	3	28
Mobilization of the sigmoid	0	1	0	4	1	1	3	10
Exposition of the ureter	0	0	0	2	1	1	3	7
Mobilization of the left flexure	0	0	0	1	0	0	2	3
Dissection of the mesentery/artery dissection	0	0	0	2	0	0	3	5
Preparation of the upper rectum	0	0	0	1	0	0	2	3
Stapling of the upper rectum	0	0	0	3	0	2	2	7
Mini laparotomy and evisceration	1	1	3	4	0	1	3	13
Resection of the sigmoid	0	1	0	3	0	2	3	9
Anastomosis	2	0	2	5	7	8	2	26
Abdominal closure	4	3	4	11	8	8	3	41

	Postgraduate year							Total
	1	2	3	4	5	6	6+	
(C)								
Thyroid resection (THYR)	54	28	35	103	60	195	31	506
No resident present								282
Resident present but no involvement	38	25	17	42	24	100	5	95
Procedure performed by resident	0	0	1	15	14	43	11	17
Sub-steps performed	16	3	17	46	22	52	15	171
Access to the thyroid gland	2	2	10	24	9	22	10	79
Mobilization of the thyroid lobe	1	0	5	13	7	22	5	53
Preparation of vagal nerve for neuromonitoring	1	0	3	12	2	21	4	43
Preparation of recurrent nerve	0	0	1	8	2	15	1	27
Preparation of parathyroids	1	0	3	11	3	12	4	34
Resection of the thyroid lobe	1	0	3	7	3	15	3	32
Closure of access	15	3	16	45	18	44	15	156

	Postgraduate year							Total
	1	2	3	4	5	6	6+	
(D)								
Minimally invasive hernia repair (MIHR)	99	62	106	109	112	78	34	600
No resident present								148
Resident present but no involvement	69	45	40	36	40	23	5	258
Procedure performed by resident	1	0	27	42	37	40	25	172
Sub-steps performed	29	17	39	31	35	15	4	170
Access to the abdomen/preperitoneal area	12	13	28	21	25	10	2	111
Identification and reposition of hernia content	1	3	12	21	13	8	1	59
Preparation of the hernia sac	0	2	10	15	8	9	0	44
Application of hernia mesh	1	1	11	19	10	6	0	48
Peritoneal closure	0	1	10	16	12	3	1	43
Closure of access	25	14	34	30	27	6	4	140

	Postgraduate year							Total
	1	2	3	4	5	6	6+	
(E)								
Pylorus-preserving pancreaticoduodenectomy (PPPD)	20	6	14	12	29	33	5	119
No resident present								61
Resident present but no involvement	12	1	7	4	12	5	0	41
Procedure performed by resident	0	0	0	0	0	0	0	0
Sub-steps performed	8	5	7	8	17	28	5	78
Laparotomy	4	4	7	5	10	24	3	57
Opening of bursa omentalis	0	0	1	3	5	16	2	27
Preparation (Kocher’s maneuver)	0	0	1	3	3	12	1	20
Lymphadenectomy	0	0	0	1	0	2	0	3
Cholecystectomy	5	2	5	6	9	14	1	42
Resection of the pancreas	0	0	0	0	0	0	0	0
Pancreatico-jejunostomy/-gastrostomy	0	0	0	0	0	0	0	0
Bilio-digestive-anastomosis	1	0	0	0	0	8	1	10
Duodeno-/gastro-jejunostomy	5	5	3	4	11	17	2	47
Abdominal closure	1	3	4	2	13	15	3	41

Regarding SIGR, the assistance of sub-steps was generally low with *n* = 55 (25.3%) procedures (Table 3B). Parts of the preparation were performed very scarcely.

In THYR, sub-steps were performed in 171 cases (21.7%). The closure of the surgical site was performed often, while the access to the thyroid was a not so common sub-step. Preparation of the vagal nerve and the resection was performed only in few cases (Table 3C).

In 170 MIHR (22.7%), access and closure of the surgical site were the most frequently performed sub-steps. Preparation, positioning of the mesh and, if applicable, peritoneal closure were less frequent (Table 3D).

The detailed analysis of sub-steps revealed that during PPPD the access to and closure of the abdomen as well as cholecystectomy and gastro-/duodenojejunostomy were most frequently performed. Pancreatic resection or anastomosis was never performed by residents. (Table 3E)

Table 3A–E reveals that the type of performed sub-step changes throughout surgical residency. With a rising postgraduate year, the sub-steps become more difficult and that simpler procedures like LCHE are more frequently performed completely.

**Fig. 3:**
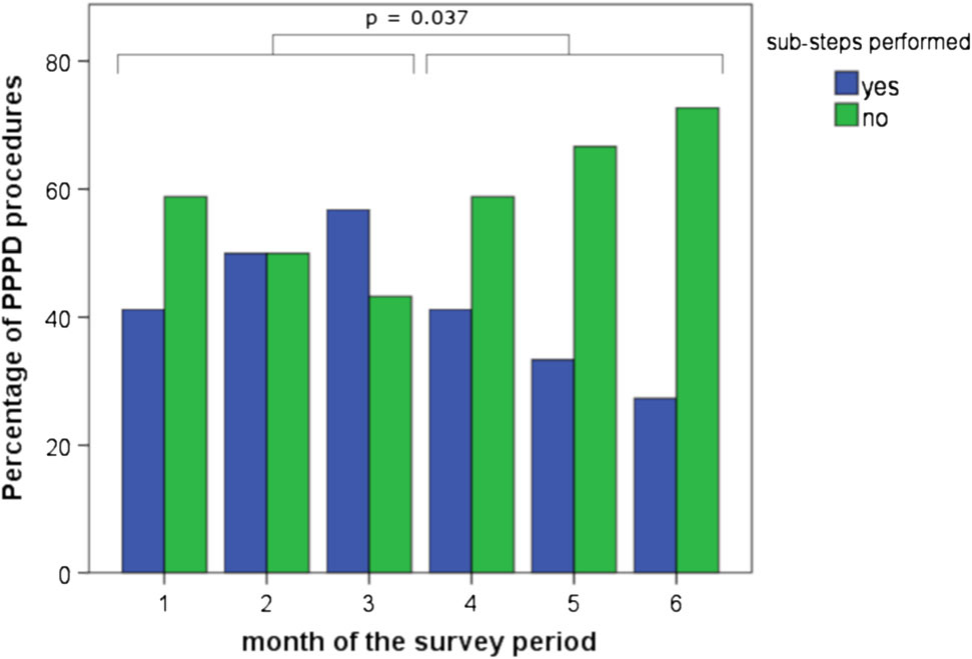
Percentage of total performed sub-steps in PPPD procedures during the study period

Under the exclusion of surgeries performed by residents, sub-steps were significantly reduced during the second half of the study period. [Months 1–3 (427/1261) vs. months 4–6 (233/985); *p* < 0.001]. Figure 3 illustrates this decline of assisted sub-steps after an initial increase exemplarily in PPPD (*p* = 0.037).

Of the 176 participants who answered the pre-study questionnaire, 107 (60%) were residents and the other 69 were fellows, attendings or the department heads. The post-study opinion survey was filled out by 112 participants, of whom 73 (65%) were residents.

In the “before study” questionnaire as well as in the “after study” survey, the participants were asked how important they find the sub-steps concept for surgical education. The majority of the participants (90.9%) found that the assistance of sub-steps was important or even very important for surgical training. After the survey, this shifted even more toward finding it important or very important (96.4%). For residents, it seems to be even more important than for fellows, attendings and the department heads (Supplementary Figure 1).

In the opinion survey, the participants were also asked, what percentage of the five signature procedures are performed completely by residents or how often sub-steps were assisted. Supplementary Figure 2 compares the real data measured during the 6 months of the survey period with the opinion data before and after the study. Before the study, the percentage of complete procedures performed by the resident was over estimated, whereas the fraction of performed sub-steps was underestimated. After the study, these estimations were more realistic in general. Interestingly, for LCHE, the extend of resident involvement was over estimated before and after the survey period. No difference in estimation of resident involvement or sub-steps was obtained between residents and others.

## Discussion

This is the first prospective analysis of the sub-step practice approach in surgical departments. With 21 participating centers of all levels of medical care in Germany, the results can be interpreted as representative. The analysis revealed that sub-steps of the selected index procedures are only performed in less than one-third of the procedures (29%), excluding the cases that were performed by the resident completely. This actually confirms the estimated number of performed sub-steps in nationwide questionnaires by the surgical societies that lay around 27% [3, 6]. The highest percentage of performed sub-steps was analyzed in PPPD with 43%. Furthermore, about 25% of the index procedures have been performed by residents. While LCHE was expectedly the most frequent resident procedure and PPPD was not performed by surgeons in training, especially the low number of sigmoid resections is alarming and supports the data gained by Kaser et al. [2], who have recently stated that fewer colonic surgeries are used for resident training. The authors also state that due to a trend toward specialization and the advancing complexity of the procedures, resident procedures are reduced. In the current analysis, residents did not perform any part of the surgery in more than half of the registered procedures (54%). This percentage may be even higher since the voluntarily participating hospitals should be considered as highly interested in surgical education in general. The exposition of surgical residents to intraoperative experience needs to be faced as one of the most important issues in surgical education in the future.

Furthermore, the assistance of training procedures and sub-steps is too low in our opinion, especially since the reason for not performing a sub-step was often due to *organizational* or *other reasons*. In these cases, no surgical resident was available during the operation in almost 80%. A recent analysis of 254 consecutive PPPD revealed that there is no difference in postoperative morbidity if the assistance is done by a fellow versus a resident [8]. A different study revealed no significant difference in postoperative complications matching 1747 thyroid surgeries with resident participation to the same number without resident involvement [9]. In an analysis by Raval et al. [10], resident involvement was even associated with a lower mortality. Hence, it is safe to perform surgeries with residents and there should always be one present in the OR. Also, it has been practiced in participating centers according to free text comments that surgical residents may be called to the OR just to perform a sub-step (e.g., laparotomy in PPPD) if the availability of the resident may not be given for the complete surgery. We hypothesize that *other reasons* are the percentage, where the surgical team is not sufficiently aware of the surgical sub-step concept. These *organizational* or *other reasons* may be influenced by a higher awareness of the concept and an appropriate coordination of the surgical team constellation.

The detailed analysis of the performed sub-steps revealed that access and closure were the most frequently assisted sub-steps in all index procedures. Without any doubt, these are the initial sub-steps in surgical training, yet especially in THYR, SIGR and PPPD, the preparational aspects need to be taught as well before advancing to the performance of the whole surgery. Like DaRosa et al. [4] have described the levels of teaching practical surgical skills should advance from “assisting” to “smart help,” to “dumb help” and finally to “performing alone.” Surgical sub-steps can be adapted to all these levels and open the possibility to establish competency-based education. This stands in contrast to a surgical training based on the simple numbers of surgeries performed by a trainee like the current board examination requirements in Germany suggest.

**Fig. 4:**
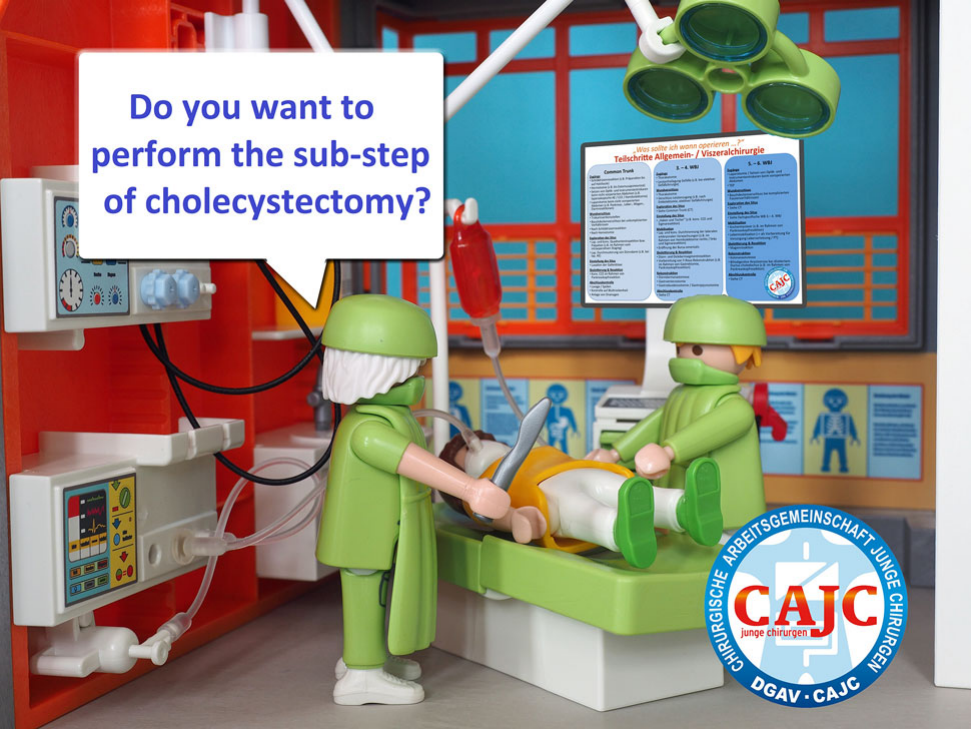
Promotion campaign of the surgical sub-step concept of the German young surgeons working group (CAJC) (Modified from [12])

The local implementation of the sub-step concept, however, needs to be promoted. The importance of the concept has been estimated “important” before and “very important” after the survey period. Especially the number of performed sub-steps is estimated more correctly in the participating centers, revealing a higher awareness of the sub-step training approach. Kneist et al. found in their analysis of the sub-step practice approach that the implementation of the concept can be successful as the percentage of performed sub-steps increased from 14 to 30% over a period of 12 months. A local presentation of the results and a regular reminding of the members of the department to assist sub-steps to residents seems to have a positive influence on the assistance of sub-steps [1, 7]. In the current analysis—where reminders were (intentionally) not performed—a significant decrease in assisted sub-steps was obtained during the second half of the survey period. Thus, we conclude that the concept needs to be promoted on a regular basis. Nationwide campaigns (Fig. 4) can only provide a certain basic awareness. The practical implementation in the surgical unit is the responsibility of the department heads, especially since the sub-step approach is one of the key aspects desired by residents to improve surgical education [11]. Furthermore, the routine performance of sub-steps in a surgical department may attract applicants and potentially improve the motivation of the whole team.

The current study has only been undertaken in Germany, which limits the transfer to other countries since surgical training differs widely throughout the world and even between European countries. Nonetheless, many aspects of our study and the surgical sub-step concept are important for other countries as well. The exclusion of patient’s with private insurance has been defined following the monocentric analysis of the concept by Kneist et al. [7]. The voluntary participation of the centers can be considered a limitation as thereby, departments with a higher interest in surgical education may be overrepresented. Thus, it can be hypothesized that the actual participation of surgical residents in the operating room is even lower.

A more detailed database like the ACS National Surgical Quality Improvement Program (NSQIP) in the US may provide information on outcome of the resident involvement in visceral surgery for Germany as well. Nonetheless, a point of criticism to the ACS NSQIP is the missing registration of the actual degree of surgical trainee participation [8]. Thus, a detailed registry also needs to include the sub-steps of a procedure to analyze the influence on operating time and patient outcome. The German Young Surgeons Working Group (CAJC) is currently planning a continuous sub-step registry in order to improve surgical training and measure the effect of the awareness campaigns. With this continuous registry, the proposed sub-step concept will hopefully be applied more often in the OR.

## Conclusion

In this first analysis of the surgical sub-step concept in German general and visceral surgery departments, the actual number of performed surgeries and sub-steps is alarming. Reasons why sub-steps are not performed are frequently organizational and may be avoidable. The current data suggest a low participation of surgical residents in the operating room, although the participating hospitals are most likely highly interested in surgical education, hence their voluntary participation. Conceptual changes and a control of surgical education are needed.

## Electronic supplementary material

Below is the link to the electronic supplementary material.

Supplementary material 1 (DOCX 149 kb)
