# Supplementary material for: See (n)One, Do (n)One, Teach (n)One: Reality of Surgical Resident Training in Germany
Source: World J Surg. 2020 Apr 30;44(8):2501–10. doi: 10.1007/s00268-020-05539-6 (PMC7326792; doi:10.1007/s00268-020-05539-6)
Supplement: Supplementary file 1 — Supplementary material 1 (DOCX 149 kb) [file 268_2020_5539_MOESM1_ESM.docx]

Supplementary figure 1

Supplementary figure 2a

Supplementary figure 2b
